# *Clostridioides difficile* in Peripartum Women: Review of Outcomes and Treatment

**DOI:** 10.3390/antibiotics14080829

**Published:** 2025-08-15

**Authors:** Ravina Kullar, Stuart Johnson, Ellie J. C. Goldstein

**Affiliations:** 1 Expert Stewardship, Inc., Newport Beach, CA 92660, USA; 2 Hines VA Hospitl Research Service, Hines, IL 60141, USA; stuart.johnson2@va.gov; 3 Chicago Stritch School of Medicine, Loyola University, Chicago, IL 60153, USA; 4 R.M. Alden Research Laboratory, Santa Monica, CA 90404, USA; ejcgmd@aol.com

**Keywords:** peripartum, *Clostridioides difficile*, CDI, fecal microbiota transplantation, probiotics

## Abstract

**Background:** *Clostridioides difficile* infection (CDI) is one of the most common healthcare-associated infections in the United States with increasing rates in younger patients and those in the community. CDI incidence may also be on the rise in peripartum women. **Methods:** We conducted a literature review to assess the incidence and outcomes of CDI in the peripartum population and review treatment options. **Results:** Peripartum patients have a high risk of complications and adverse events associated with CDI. Most patients have been treated with vancomycin or metronidazole; however, cases of patients recurring on standard treatment have been described, with patients having successful outcomes with fidaxomicin or fecal microbiota transplantation (FMT). Probiotics have been shown to be safe in peripartum women; however, the role in preventing primary and secondary CDI has not been studied. **Conclusions:** Peripartum women that develop CDI are at increased risk for complications. Treatment includes vancomycin, metronidazole, or fidaxomicin or FMT for recurrent cases.

## 1. Introduction

*Clostridioides difficile,* previously named *Clostridium difficile* is an anaerobic bacterium that exists in the environment as a spore which when ingested causes disease under favorable conditions in the human colon [1]. The host becomes susceptible when the normal microbiota is disrupted resulting in an increase in intestinal primary bile acids which facilitates germination of *C. difficile* and subsequent elaboration of toxins leading to epithelial cell disruption and diarrhea [2].

*Clostridioides difficile* infection (CDI) is a common cause of diarrheal infection worldwide, with both community and healthcare-associated acquisition. The Centers for Disease Control and Prevention (CDC) reported 101.3 cases per 100,000 people. Of these cases, 51.2% were community-acquired (CA-CDI), and 50.1% were healthcare-associated (HA-CDI). Almost half a million individuals acquire CDI every year in the United States, contributing to ~15,000 deaths each year [3]. In Europe, the mean incidence of CDI has been estimated to be 3.48 cases per 10,000 patient days, with many cases (60.9%) being HA-CDI [4]. Up to 35% of those that have a first episode of CDI end up developing a recurrent episode [5,6,7]. In those that incur a first episode of CDI, ~40–60% have a second recurrence [8], and recurrences are associated with increased morbidity [7]. Testing for CDI usually involves glutamate dehydrogenase (GDH) and toxin A&B and PCR. GDH uses antibodies to test for presence of the GDH enzyme, a protein preserved in all *C. difficile* bacteria (both toxin producers or non-toxogenic strains) and PCR uses DNA primers to amplify copies of a targeted gene, primarily the toxin B gene. However, two experimental surrogate markers are being tested as endpoints for CDI treatment success: persistence or elimination of key bacterial phyla essential for metabolism of bile salt; and measuring the primary and secondary bile acid ratios and levels.

**Figure 1:**
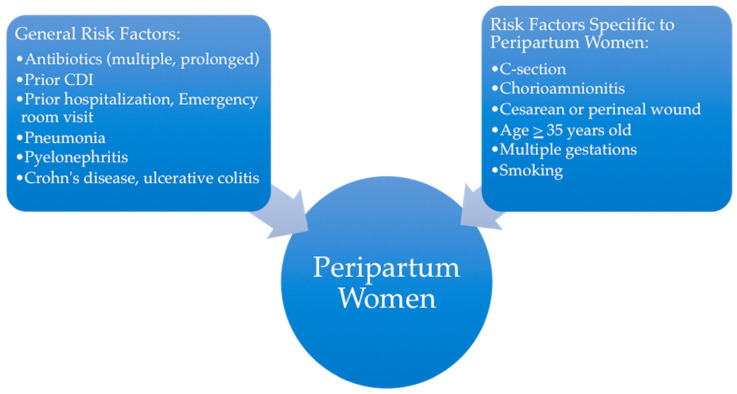
Risk factors for *C. difficile* infection (CDI) in peripartum women.

The primary risk factor for acquiring CDI is prior antibiotic exposure; the most commonly associated agents include cephalosporins, fluoroquinolones, ampicillin/amoxicillin, and clindamycin. Antibiotics disrupt normal bowel flora and promote colonic *C. difficile* overgrowth and subsequent exotoxin production. Particularly, prolonged antibiotic and multiple uses of antibiotics are associated with CDI. Other risk factors include advanced age, exposure to healthcare settings, comorbidities such as liver disease, kidney disease, and cardiovascular disease, proton pump inhibitors, and impaired humoral immunity [9]. The patient populations susceptible to CDI have now expanded to include pregnant women. A study published by the American College of Obstetricians and Gynecologists identified smoking, multiple gestations, pneumonia, pyelonephritis, cesarean section, and perineal wound infections as risk factors for CDI during pregnancy [10]. Pregnant women are considered immunocompromised hosts by virtue of immune adaptation to their allogeneic fetus which may also influence CDI risk. Figure 1 displays risk factors for the general population acquiring CDI and those specific to peripartum women. Few studies evaluate the outcomes of CDI in the peripartum patient population.

Recently we were involved in the management of a colleague who developed CDI in the later stages of her pregnancy (a formal consent was received). At 32 weeks gestation she developed pneumonia and was treated with cefdinir, azithromycin, albuterol inhaler, and prednisone. One month later, she developed fever and loose stools and was admitted to the hospital. Stool *C. difficile* testing by GDH and toxin A&B and PCR were all positive. She was then treated with oral vancomycin (125 mg qid) for 10 days. She delivered a healthy baby vaginally at 36 weeks gestation with no other major complications. She was discharged 5 days post-delivery and completed her vancomycin course at home in addition to a course of probiotics (Bio-K+, 10 billion per day po; Kerry Group, Sainte-Claire, QC, Canada). She subsequently had 2 recurrent CDI episodes, the first recurrence occurring one week after completing vancomycin and was treated with fidaxomicin for 10 days. The second recurrence occurred 2 weeks post-completion of fidaxomicin and was transient not requiring treatment. Probiotics were continued for 6 months since the first episode of CDI. Herein, we review the incidence, outcomes, and treatment of CDI in the peripartum patient population.

## 2. Search Methodology

A literature review was conducted using the PubMed, Embase, and Researchgate databases up to 1 July 2025, aiming to identify relevant publications addressing *C. difficile* in the peripartum patient population. The following search strings were used: “peripartum”, “*Clostridium difficile*”, and “*Clostridioides difficile*”. All selected articles were further screened for additional relevant articles through their reference lists. Publications were eligible for inclusion if they met the following criteria: included peripartum women with CDI and ≥1 of the following: (1) described demographics of these patients; (2) assessed outcomes; and/or (3) described treatment course. Only publications in English were included.

## 3. Results

### 3.1. Pregnancy and Immune Status

During pregnancy there is a modulation of the immune system. The placenta and the fetus embody an additional immunological organ which impacts the overall response of the mother to microbial infections. During pregnancy, studies have indicated that complement activity increases, particularly C3a, C4a, C5a, C4d, C3a, C3, C9 plasma levels and the Serum Complement Membrane Attack Complex SC5b9 being increased [11,12]. Additionally, in pregnant women, the Th1 response is replaced by a Th2 response. *C. difficile*-induced colitis comprises neutrophils entering the colonic mucosa, which is mediated by a Th1 immune response. Studies have shown that the immunoregulation correlated with pregnancy increases CDI risk by downregulating the Th1 response that is required to control the disease [13]. This is partly due to an increase in cortisol, estrogen, and progesterone levels, leading to an anti-inflammatory response. Animal models have shown that this interaction between *C. difficile* toxin and hormones may partly explain disease susceptibility and outcome of peripartum CDI [14]. Pregnant women also often have decreased IgG levels, especially in the 2nd and 3rd trimesters, which can affect their CDI immunity.

### 3.2. Peripartum Women and CDI Cases

In April 2006 to 2007, an outbreak of 20 cases of CDI in peripartum women was reported at the University of Washington with an incidence of 7.5 cases/1000 deliveries [15]. Risk factors included C-section (*p* = 0.003) antibiotic use (*p* = 0.001) and chorioamnionitis (*p* = 0.001). Three cases developed CDI during a separate prenatal admission and seven patients were diagnosed during their delivery hospitalization. Ten postpartum cases were diagnosed after being discharged from the hospital, with eight of these women having to be readmitted to the hospital. While sporadic cases continued to occur, the institution of infection prevention and antimicrobial control measures seemed to have controlled the outbreak. Use of combination antibiotics remained a significant independent risk factor for CDI in the multivariate analysis. Meda et al. reported a cluster of cases among pregnant/postpartum women in two neighboring secondary care hospitals in South-East England [16]. Eight of the 10 cases presented after hospital discharge and all had received broad-spectrum antibiotics. Potential environmental vectors that were difficult to decontaminate were identified. An infection control care bundle was implemented which may have resolved the outbreak.

Using a national database, Ruiter-Ligeti et al. reported 0.2% (2757/13,881,592) of live births (1999–2013) were complicated by CDI. During their study period the rate doubled from 15 to 30 cases/1000 live births *p* < 0.001) [10]. They noted that risk factors associated with developing CDI included “age older than 35 years, multiple gestations, smoking, Crohn’s disease, ulcerative colitis, long-term antibiotic use, pneumonia, pyelonephritis as well as cesarean or perineal wound infection.” More importantly, CDI cases had a greater risk of “maternal mortality (8.0/1000 vs. 0.1/1000, sepsis (46.4/1000 vs. 0.6/1000, adjusted OR 59.1, 95% CI 48.8–71.6), paralytic ileus (58.0/1000 vs. 1.5/1000, adjusted OR 33.1, 95% CI 27.5–39.8), venous thromboembolism (38.4/1000 vs. 3.1/1000, adjusted OR 8.1, 95% CI 6.5–10.2)”, and prolonged hospital stays greater than 2 weeks (173.0/1000 vs. 6.5/1000, adjusted OR 24.3, 95% CI 21.6–27.4). Venugopal et al. performed a single hospital retrospective chart review of patients from 2002 to 2007 to determine CDI incidence in peripartum women [17]. They reported 12 cases leading to an incidence of 0.7 cases/1000 obstetric ward admissions 92% of which had prior antibiotics and 67% were healthcare-related. While the spectrum of CDI disease was similar in pregnant and non-pregnant patients, older age was a risk factor. Cesarean section patients had a higher rate of CDI compared to patients undergoing vaginal delivery (2.2 vs. 0.2/1000) live births.

Saha et al. assessed the incidence and risk factors of peripartum CDI in a single-center retrospective cohort study conducted from 1997 to 2017 [18]. Out of 125, 683 pregnancies, 80 cases of peripartum CDI (47 during pregnancy, 33 postpartum) were identified, with the median age being 27 years (range 20–41) and six patients having a history of acquiring CDI in the past. The incidence of CDI increased 3.4-fold (95% confidence interval 1.5–7.4, *p*  =  0.005). The most common risk factors within the 90 days prior to the CDI episode included outpatient or emergency room visit (83%), systemic antibiotics (70%), and hospitalization (61%). The Emerging Infections Network (EIN), which includes over 900 infectious diseases clinicians worldwide, surveyed clinicians to estimate if CDI is increasing in peripartum women [19]. Those that were surveyed were asked if they had “seen” or were “aware of” CDI cases, the timing of these cases in relation to delivery, and complications associated with CDI. Four hundred and nineteen out of 798 (52.5%) physicians responded, with 37 (8.8%) that had “seen” or were “aware of” a total of 55 cases of peripartum CDI over 6 months. Ten women with severe CDI were described in further detail, with the mean age being 28.2 (mean 18–40) years. Three patients had a prior history of hospitalization and 9 had previous antibiotic use in the 3 months preceding their admission for CDI. Six cases happened prior to delivery and 3 out of 4 postpartum cases occurred within 1 week of delivery. Four cases of peripartum CDI were described in detail from Texas Medical Center [20]. Two of the 4 patients were infected with the hypervirulent strain, defined as *tdC* deletion and presence of the binary toxin. All of the cases had a history of prior antibiotic use, and two patients had a history of hospitalization prior to CDI onset.

Ye et al. isolated *C. difficile* in 37/1009 (3.7%) of pregnant Chinese women compared to 1.4% (9/651) non-pregnant women [21]. Of those isolates that were *C. difficile* positive, 27% (N = 10) were toxigenic isolates containing both toxin A and toxin B genes (A+B+), 13.5% (N = 5) contained the toxin B gene (A−B+) only, and 59.5% (N = 22) were non-toxigenic isolates. Multilocus sequence typing (MLST) showed that the sequence types ST-37 (ribotype (RT) 017) and ST-54 (RT 012) were the most frequent toxigenic types observed in pregnant women. Ferraris et al. found that 52% of colonized neonates harbored RT 062 (35%) and RT 038 (60%) [22].

In France, Rousseau et al. followed 10 healthy infants for 1 year after birth and reported that all infants became colonized with *C. difficile* [23]. In a point prevalence study of two day- nurseries that 45% carried *C. difficile* including 13% with toxigenic strains. In another French study eight antepartum and six post-partum CDI cases were retrospectively studied in six French centers (2008–2013) [24]. Prior antimicrobial therapy, C-section and underlying disease were again found as risk factors. A one-year longitudinal study of *C. difficile* carriage in preterm neonates found 52% ((199/378) became culture positive [22]. Higher rates were found in neonates with higher gestational age (*p* = 0.006) and higher birthweights (*p* = 0.016).

### 3.3. Peripartum Women and CDI: Outcomes

Outcomes were described in an outbreak of 20 peripartum CDI cases [15]. Eighteen of these cases had obstetric complications with some having multiple complications, including preterm labor; preterm, premature rupture of membranes; chorioamnionitis; postpartum endometritis; pyelonephritis; gestational hypertension; placenta previa; abruption; mastitis; pneumonia; fetal anomalies; and pulmonary embolus. Eight patients were readmitted to the hospital postpartum due to diarrhea and fever. These patients incurred a total of 22 extra inpatient days or an average of almost 3 extra days per case. One patient developed septic shock and toxic mega colon, but no deaths occurred in this series. Five cases had a gestational age at delivery ≤ 30 weeks (range 23–30 weeks).

Rouphael et al. described the outcomes in 10 cases of pregnant women with severe CDI [19]. Most common symptoms included abdominal pain (N = 9), diarrhea (N = 7), and distension (N = 6). The median hospital stay was 15 days (range 2–80). One patient was diagnosed with CDI during the autopsy and never received any treatment. Several patients were admitted to the ICU (6 of 10 patients) and five patients received a subtotal colectomy. Many experienced complications: 6 patients developed toxic mega colon, 3 had sepsis, 3 had renal failure, and 2 had disseminated intravascular coagulation (DIC). CDI recurrence occurred in two patients. Death occurred in 3 patients and there were 3 stillbirths.

Pregnancy and neonatal outcomes in peripartum women that acquired CDI were evaluated by Saha et al. in 67 matched pairs with CDI before and during pregnancy [25]. Over the course of twenty-one years (1997–2018), 101 cases and 100 controls were included. CDI-related complications were shock (N = 8), sepsis (N = 4), ICU admission (N = 3), and colectomy (N = 2). Cases had higher odds of cesarean delivery (*p* = 0.02) and lower odds of Group B *Streptococcus* (GBS) infection/colonization (*p* = 0.03). After controlling for labor arrest disorders, the odds of cesarean delivery remained high [odds ratio (OR): 17.23 (95% confidence interval (CI), 2.19–543.19; *p* = 0.004)]; odds of GBS remained low after controlling for antibiotic use (OR: 0.25, 95% CI, 0.04–0.99; *p* = 0.049). No differences were noted in neonatal outcomes between cases and controls.

In another study where delivery admission records of pregnant women were reviewed between 1999 and 2013 from a nationwide database, of the total 13,881,592 births, 2757 (0.02%) admissions had concomitant CDI [10]. Those diagnosed with CDI during pregnancy were correlated with a significant increase in maternal death (8.0/1000 vs. 0.1/1000, adjusted odds ratio [OR] 56.8, 95% CI 35.8–90.1), increase in sepsis (46.4/1000 vs. 0.6/1000, adjusted OR 59.1, 95% CI 48.8–71.6), paralytic ileus (58.0/1000 vs. 1.5/1000, adjusted OR 33.1, 95% CI 27.5–39.8), venous thromboembolism (38.4/1000 vs. 3.1/1000, adjusted OR 8.1, 95% CI 6.5–10.2), and hospital stays > 2 weeks (173.0/1000 vs. 6.5/1000, adjusted OR 24.3, 95% CI 21.6–27.4).

### 3.4. Treatment Options

Therapy for pregnant patients with CDI is far from standardized. The two main antibiotics for treating CDI, vancomycin and fidaxomicin are minimally absorbed which may decrease risk for potential complications, but they have not been extensively studied in pregnancy and are listed as Pregnancy Category B. In their retrospective cohort study, Saha et al. noted that the most common initial treatment was metronidazole in 66.3% (N = 65), followed by vancomycin in 29.6% (N = 29) of patients [25]. Overall, 24.7% of patients required treatment to be changed (metronidazole vs. vancomycin: *p* = 0.39). Of these, 17 were due to non-resolution of diarrhea and 7 due to adverse events (nausea most common). About 3% (N = 3) of patients were refractory to all antibiotic treatment for CDI; one patient received fecal microbiota transplantation (FMT) post fidaxomicin and two patients received a colectomy. Eleven patients received recurrent CDI (within 56 days of completing treatment); FMT was administered to five patients for recurrent CDI, with all FMT procedures were performed via colonoscopy. Saeedi et al. described a case of a 28-year-old pregnant woman with recurrent CDI in spite of vancomycin and fidaxomicin treatment [26]. At 18 weeks of gestation the patient underwent successful FMT through colonoscopy and had a term vaginal delivery at 39 weeks of gestation. At the 4-month follow-up post-delivery, the infant was developing normally, and no further recurrence of CDI was reported. In Rouphael et al.’s case series of severe CDI peripartum cases, vancomycin with metronidazole was given to most patients (6 of 10 patients) [19].

## 4. Discussion

Pregnant women are likely at increased risk for CDI particularly during the peripartum period despite their relatively younger age in comparison to those typically at risk for CDI. Most deliveries in the U.S. and other developed countries occur in hospitals, a setting known for increased exposure to CDI, antibiotics are frequently administered to pregnant women around the time they give birth, and some undergo surgical procedures such as cesarean section. In addition, pregnant women are relatively immunosuppressed. This increased risk appears reflected in our review which includes retrospective studies of CDI incidence, outbreaks, and outcomes among peripartum women in the U.S., Europe, and China. In the case where a colleague developed CDI while she was pregnant, her risk factors included a history of pneumonia and a course of antibiotics one month prior to acquisition of *C. difficile.* The manifestations of CDI in peripartum women reflect the spectrum seen in non-pregnant patients including reports of severe disease and death but also include obstetrical complications. Treatment of CDI in peripartum women is not yet standardized, but there are multiple reports of successful treatments with vancomycin, metronidazole, fidaxomicin, and FMT. Safety of these treatments is not fully assessed, and long-term maternal and child complications need to be assessed.

Probiotics for CDI have been extensively studied in general. McFarland et al. found that not all probiotics have efficacy for antibiotic associated diarrhea (AAD) and CDI and efficacy is strain-specific [27]. Clinical practice guidelines for the use of probiotics in the primary prevention of CDI vary. Four probiotics have been recommended by the American Gastroenterology Association guidelines for the prevention of CDI (*Saccharomyces boulardii* CNCM I-745 or a three-strain blend of *Lactobacillus acidophilus* CL1285 and *Lactobacillus casei.* LBC80R and *Lactobacillus rhamnosus* CLR2 or a three-strain blend of *L. acidophilus*, *Lactobacillus bulgaricus*, and *Bifidobacterium bifidum* or a four-strain blend of *L. acidophilus*, *L. bulgaricus*, *B. bifidum*, and *Streptococcus thermophilus* [28]. However, the American College of Gastroenterology guidelines and the European Society of Clinical Microbiology and Infectious Diseases (ESCMID) guidelines do not believe there is enough data to support for probiotics for the primary prevention of CDI [29,30,31]. McFarland et al. noted that prior reviews and meta-analyses reviewing phase 2 and phase 3 trials did not show a strong correlation of use of probiotics for secondary prevention of CDI [28,32]. There has been a significant decrease in CDI recurrences with some probiotics (*S. boulardii* CNCM I-745 and the three-strain *Lactobacilli* blend (Bio-K+) [33]. According to the American Gastroenterology Association (AGA), a single-strain probiotic (*S. boulardii* CNCM I-745, based on nine trials) and three different multi-strain blends (only the three-strain blend of *L. acidophilus* CL1285, *Lbc. casei* LBC80R, and *Lbc. rhamnosus* CLR2 had multiple RCTs) are recommended for the prevention of CDI recurrence [28]. However, the American College of Gastroenterology (ACG) does not recommend probiotics [29]. The use of probiotics for primary and secondary CDI prevention in peripartum patients has not been studied but is warranted. Probiotics appear to be safe in pregnant and lactating women, with the current literature not indicating an increase in adverse pregnancy outcomes [34,35].

## 5. Conclusions

Peripartum women that develop CDI are at increased risk for complications. Treatment includes vancomycin, metronidazole, or fidaxomicin or FMT for recurrent cases. The role of probiotics in primary and secondary prevention of CDI in peripartum patients needs further evaluation.
